# Effect of Postoperative High-Concentration Oxygen on Recovery After Thyroid Surgery: A Prospective, Open, Randomized, Controlled Study

**DOI:** 10.3389/fendo.2021.595571

**Published:** 2021-07-05

**Authors:** Qian Zhou, Ming Cai, Juxiang Gou, Ning Ning

**Affiliations:** ^1^ Department of Thyroid Surgery, West China Hospital, Sichuan University/West China School of Nursing, Sichuan University, Chengdu, China; ^2^ West China School of Nursing, Sichuan University/Department of Orthopedics, West China Hospital, Sichuan University, Chengdu, China

**Keywords:** high-concentration oxygen, thyroid surgery, pain, postoperative nausea and vomiting, voice changes, swallowing function, parathyroid function

## Abstract

**Objective:**

To investigate the effectiveness of high-concentration oxygen on the improvement of throat symptoms and voice changes after thyroid surgery and its protection of the parathyroid function.

**Methods:**

A total of 600 patients undergoing thyroid surgery who met the inclusion criteria were randomly divided into the experimental group (n = 300) and the control group (n = 300) by using a random number generator. The patients in the experimental group underwent high-oxygen treatment [FiO_2_80 (8 L/min)] for 6 continuous hours after returning to the ward after surgery. The patients in the control group underwent low-oxygen treatment [FiO_2_30 (2 L/min)] for 6 continuous hours after returning to the ward after surgery.

**Results:**

The postoperative incision pain score of patients in the experimental group was significantly better than that in the control group at 6 h (1.07 ± 0.80 *VS* 1.23 ± 0.95, *P*=0.031) and 12 h (1.08 ± 0.64 *VS* 1.20 ± 0.73, *P*=0.041). The postoperative throat pain score of the patients in the experimental group was significantly better than that of the control group at 6 h (1.40 ± 0.85 *VS* 1.59 ± 0.97, *P*=0.011) and 12 h (1.40 ± 0.85 *VS* 1.59 ± 0.97, *P*=0.019). The PONV score of the patients in the experimental group was significantly better than that of the control group at 12 h (0.09 ± 0.19 *VS* 0.14 ± 0.37, *P*=0.024). The Voice Handicap Index score of the patients in the experimental group was significantly better than that of the control group at 24 h (2.89 ± 5.92 *VS* 4.10 ± 6.31, *P*=0.017), 36 h (2.46 ± 5.06 *VS* 3.43 ± 5.97, *P*=0.035), and 48 h (2.46 ± 5.06 *VS* 3.43 ± 5.97, *P*=0.032).

**Conclusion:**

High-concentration oxygen can alleviate PONV and pain after thyroid surgery, with less severe voice changes potentially. However, its effects on swallowing function, and parathyroid function need to be further verified.

**Clinical Trial Registration Number:**

ChiCTR-IOR-17012765 (China Clinical Trial Registry clinical trial registration center [http://www.chictr.org.cn/index.aspx)

## Introduction

In recent years, the number of patients with thyroid cancer has exploded, making it the solid malignant tumor with the fastest-growing incidence (1). Surgery is the main method of treating thyroid cancer (2). In view of the large increase in the number of patients in recent years, thyroid surgery has become one of the most common surgical procedures.

Although thyroid surgery is considered to be a safer surgical procedure, various complications after surgery cannot be ignored. The blood vessels and nerves around the thyroid glands are abundant and are closely related to the oropharynx, esophagus, and trachea. A variety of throat symptoms, such as postoperative nausea and vomiting (PONV), pain and difficulty swallowing, can appear after surgery. Their incidence is reported to be 20-30% to 70-80% (3, 4). At the same time, transient recurrent laryngeal nerve and superior laryngeal nerve injury during operation can lead to aspiration of thin liquids and voice changes (5). Some 30-87% of patients may still present with minor transient voice changes even without obvious nerve damage (6–8). Surgery can also affect the blood supply to the parathyroid glands and even require autologous transplantation, resulting in hypoparathyroidism and numbness or paresthesias in the hands, feet, mouth (9). It is reported that the incidence rate of temporary hypoparathyroidism is 14-60% after thyroidectomy (2). These symptoms may lead to delays in the patient’s postoperative recovery (10).

The current prevention and treatment methods for complications after thyroid surgery include adjusting intraoperative anesthesia protocols, applying standardized surgical methods during surgery, and developing new surgical techniques. High-concentration oxygen has been used in the prevention and treatment of various surgical complications. A number of randomized controlled trials have shown that giving high-concentration oxygen during abdominal surgery, gynecological-obstetric surgery and tonsil surgery can effectively improve symptoms such as postoperative nausea and vomiting (11–13) and reduce the incidence of surgical incision infections (14–17). There is only one report on the effect of high-concentration oxygen on recovery after thyroid surgery (18), but the study only included patients with benign thyroid lesions and people in Europe and the United States. Moreover, the experimental designs have needed improvement and their samples were small.

This prospective, open, randomized, large-scale clinical trial was the first conduced in mainland China to investigate the effects of high-concentration oxygen in reducing throat symptoms and voice changes in patients with thyroid surgery and protecting the functions of the parathyroid function.

## Materials and Methods

From April 2019 to November 2019, we conducted this prospective, open, randomized, controlled study (China Clinical Trial Registry clinical trial registration center [http://www.chictr.org.cn/index.aspx] Registration Number: ChiCTR-IOR-17012765) in patients who underwent thyroid surgery at the Department of Thyroid Surgery, West China Hospital, Sichuan University. This research protocol had been approved by the Ethics Committee on Biomedical Research, West China Hospital of Sichuan University. The number of participants was proposed to be 600, with 300 for each group (experimental *vs.* control group, 1:1). The inclusion criteria were (1) patients aged between 18 and 80 years; (2) patients with thyroid papillary carcinoma who had chosen surgical treatment; (3) patients undergoing general anesthesia for the surgery. The exclusion criteria were (1) patients in which lateral cervical lymph node dissection was expected; (2) patients with a history of neck lesion treatment (such as thyroid or other neck surgery history or radioactive iodine treatment history); (3) patients who had experienced a voice change before surgery or in whom laryngoscopy confirmed the diagnosis of a lesion that can cause voice changes (such as vocal cord paralysis, vocal cord thickening, vocal cord polyps, and vocal cord nodules); (4) patients who had difficulties in language communication (communication difficulties); (5) patients who had conditions or symptoms that would affect the evaluation of our outcomes (such as a history of vomiting, history of pain, or the use of antiemetics, analgesics 48 h before surgery); (6) patients with pulmonary dysfunction; (7) patients accompanied by other malignancies; and (8) patients who refused to participate in this study. The censor criteria were (1) patients who failed to complete the oxygen treatment according to the protocol of the grouping; (2) patients who did not complete oxygen treatment using the face mask; (3) patients who did not complete the postoperative evaluation; (4) patients with placement of an analgesia pump after surgery.

After screening patients according to the inclusion and exclusion criteria, all patients voluntarily signed the informed consent to participate in the study after they fully understood the study, met for a question-and-answer session, and underwent training on how to complete the assessment scale. A random number generator was used to generate a random number table. Consecutive patients were enrolled in the experimental group or control group according to the random number table before surgery. The random number table was input by the data administrator into a separate Excel spreadsheet and saved. On the day of the surgery, the data administrator gave the patient’s grouping information to the nurse in charge, who completed the oxygen treatment when the patient had returned to the ward after the surgery. Patients in the control group received low-concentration oxygen treatment: the patient was administered low-concentration [FiO_2_30 (2 L/min)] *via* nasal cannula for 6 h after returning to the ward, and the oxygen treatment was discontinued after 6 h. Patients in the experimental group received high-concentration oxygen treatment: the patient was administered high-concentration [FiO_2_80 (8 L/min)] *via* a sealed mask for 6 h after returning to the ward, and the oxygen treatment was discontinued after 6 h. Both groups of oxygen are humidified. We arranged the two groups of patients in different wards.

The data collectors and statistical analysts were blinded to the study details. All patients underwent general anesthesia with the same protocol: propofol for sedation, sufentanil or remifentanil for intraoperative sedation and analgesia, atracurium for muscle relaxation, sevoflurane for intraoperative anesthesia maintenance, and neostigmine as an antagonist of the muscle relaxant to relieve muscle relaxation. All surgeries followed standard surgical procedures: thyroid lobectomy/total thyroidectomy plus unilateral/bilateral node lymph node dissection in the central compartment of the neck, routine intraoperative neuroelectrophysiological monitoring to evaluate the recurrent laryngeal nerve function during surgery, and routine use of plasma drainage tube after operation, and postoperative pain management without patient-controlled analgesia pump.

The World Health Organization (WHO) standard (0-3 points) was used to evaluate the severity of PONV (19). Pain evaluation included throat pain and surgical incision pain. A visual analog scale (0, no pain to 10, intolerable pain) was used for pain assessment (20). The water swallow test was used to evaluate the degree of difficulty swallowing (21): profile 1: the patient could drink all the water in 1 gulp without choking; profile 2: the patient could drink all the water in 2 or more gulps without choking; profile 3: the patient could drink all the water in 1 gulp, but with some choking; profile 4: the patient often choked and had difficulty drinking all the water. The presence or absence of numbness or convulsions of the hand, foot, and perioral area in the patient was recorded. The Simplified Chinese Version Voice Handicap Index (VHI) scale and Voice-Related Quality of Life (V-RQoL) scale (22) were used to assess subjective voice changes. The evaluation time points of the above indicators were the day before surgery and 6 h, 12 h, 24 h, 36 h, and 48 h after returning to the ward. Serum parathyroid hormone (PTH) and blood calcium levels before and after surgery were determined to assess changes in parathyroid function. The assessment included subjective discomfort (numbness or paresthesias of the hand, foot, and perioral area) and objective parathyroid function (serum PTH and blood calcium). Sociodemographic data were also collected on the day of admission. The basic clinical data were collected on the first day after surgery. The incision healing, postoperative drainage amount, and postoperative length of hospital stay were recorded on the day of discharge. All data were independently evaluated, recorded, and summarized by nurses trained in assessment methods.

### Statistical Analysis

Statistical analysis was performed using SPSS 20.0 (SPSS Inc., Chicago, IL, USA). The qualitative data with a normal distribution are expressed as mean ± standard deviation. The quantitative data are expressed as frequency and percentage. The qualitative data with a normal distribution and homogeneity of variance were compared using the independent-sample t test. The quantitative data were compared using the chi-squared test or rank sum test. The two-independent-sample t-test or rank-sum test was used to compare variables of the outcomes, including PONV, pain, and voice changes, between the two groups. P < 0.05 was considered statistically significant.

## Results

**Figure 1:**
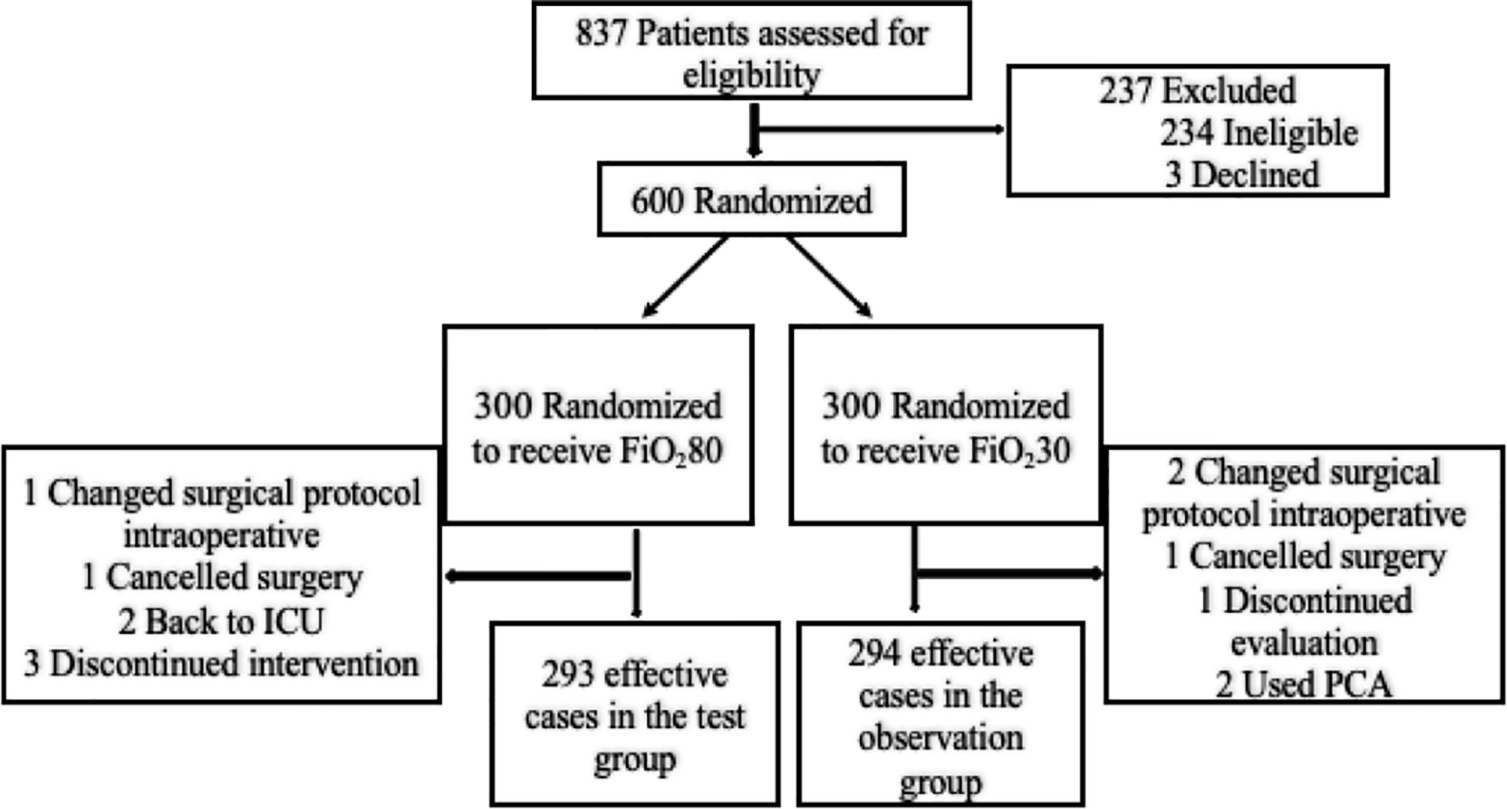
CONSORT Flow Study Diagram.

As showed in **Figure 1**, of the 837 patients evaluated, 600 (71.7%) patients who met the inclusion criteria were included in the study after signing the informed consent and were grouped randomly. The experimental group received high-concentration oxygen treatment (n = 300), and the control group received low-concentration oxygen treatment (n = 300). A total of 13 patients were censored during the study, including 7 patients in the experimental group (one patient underwent lymph node dissection in the lateral compartment of the neck, one patient was removed due to cancellation of surgery, two patients were treated in the ICU after surgery, and three patients withdrew from the study), and 6 patients in the control group (one patient underwent lymph node dissection in the lateral compartment of the neck, one patient was removed due to cancellation of surgery, one patient did not complete the evaluation, and two patients were treated with an analgesia pump after surgery). The final number of effective cases was 587: 293 cases in the experimental group and 294 cases in the control group. There was no significant difference in the withdrawal rate between the two groups. The overall withdrawal rate was 2.2%. The study flowchart is shown in **Figure 1**. As showed in **Table 1**, there were no significant differences in sociodemographic characteristics or basic clinical data between the two groups of patients (P > 0.05). The average age of patients was 41.88 ± 11.10 years. Of the patients, 453 patients were female (77.17%). There were no significant differences in the length of hospital stay (2.90 ± 1.08 *vs*. 2.97 ± 0.99) or postoperative incision drainage amount [80 (55,110) *vs.* 80 (62,107)] between the two groups (P > 0.05). No incision bleeding or reoperation was reported during the hospitalization period. Incision infection (n = 5) and incision fat liquefaction (n = 1) were reported after discharge. None of these adverse events were determined to be related to the trial. The incidence of adverse events was similar between the two groups (3 *vs*. 3). Oxygen saturation during the placement of the ECG monitor in both groups was within the normal range.

**Table 1:** Baseline data of the patients.

Variables	Experimental group (n = 293)	Control group (n = 294)	Statistics	*P*
Age (years)	41.35±10.72	42.40±11.47	*t* =1.138	0.256
Sex			*χ^2 =^*1.446	0.229
Male	73 (24.91%)	61 (20.74%)		
Female	220 (75.09%)	233 (79.26%)		
Ethnicity			*χ^2 =^*0.500	0.725
Han ethnicity	290 (98.98%)	289 (98.30%)		
Minority ethnicity	3 (1.02%)	5 (1.70%)		
Height (m)	1.62 ± 0.76	1.62 ± 0.73	*t* =1.356	0.176
Weight (kg)	61.18 ± 11.07	60.87 ± 10.77	*t* =0.352	0.725
Body Mass Index (BMI)	23.08 ± 2.99	23.24 ± 3.29	*t* =0.610	0.542
History of alcohol use			*χ^2 =^*0.024	0.877
Yes	28 (9.56%)	27 (9.18%)		
No	265 (90.44%)	267 (90.82%)		
History of tobacco use			*χ^2 =^*1.659	0.198
Yes	32 (10.92%)	23 (7.82%)		
No	261 (89.08%)	271 (92.18%)		
TSH	2.55 ± 3.30	2.58 ± 1.67	*t* =0.143	0.784
FT_3_	4.94 ± 0.96	4.88 ± 1.08	*t* =0.732	0.762
FT_4_	17.93 ± 3.27	17.76 ± 7.78	*t* =0.340	0.426
Hyperthyroidism			*z* =0.895	0.371
Yes	4 (1.37%)	7 (2.38%)		
No	289 (98.63%)	287 (97.62%)		
Hypothyroidism			*z* =0.010	0.992
Yes	2 (0.68%)	2 (0.68%)		
No	291 (99.32%)	292 (99.32%)		
Surgery range			*z*=0.633	0.527
LT+UCND	132 (45.05%)	128 (43.54%)		
TT+UCND	13 (4.44%)	12 (4.08%)		
TT+BCND	148 (50.51%)	154 (52.38%)		
Number of transplanted parathyroid glands			*z* =1.211	0.226
0	66 (22.53%)	74 (25.17%)		
1	169 (57.68%)	173 (58.84%)		
2	56 (19.11%)	46 (15.65%)		
3	2 (0.68%)	1 (0.34%)		
Maximum diameter (mm) of tumor	13.76 ± 9.19	13.70 ± 9.36	*t* =0.074	0.735
Tumor Characteristics				
T0	3 (1.02%)	9 (3.06%)	*z*=0.501	0.617
T1	260 (88.74%)	254 (86.40%)		
T2	15 (5.12%)	11 (3.74%)		
T3	13 (4.44%)	14 (4.76%)		
T4	2 (0.68%)	6 (2.04%)		
N0	201 (68.60%)	191 (64.97%)	*z*=0.693	0.488
N1	23 (7.85%)	32 (10.88%)		
Nx	69 (23.55%)	71 (24.15%)		
Tumor staging			*z*=0.187	0.852
I	279 (95.22%)	280 (95.24%)		
II	14 (4.78%)	12 (4.08%)		
III	0	2 (0.68%)		

LT, lobectomy of thyroid; TT, total thyroidectomy; UCND, unilateral central neck lymph node dissection; BCND, bilateral central neck lymph node dissection; T, tumor; N, node; M, metastasis.

The postoperative incision and throat pain scores in the experimental group were significantly better than those in the control group (P < 0.05) at 6 h (1.07 ± 0.80 *vs*. 1.23 ± 0.95) and 12 h (1.08 ± 0.64 *vs*. 1.20 ± 0.73) after returning to the ward (**Table 2**). The PONV score of patients in the experimental group was significantly better than that in the control group (P < 0.05) at 12 h after returning to the ward (0.09 ± 0.19 *vs*. 0.14 ± 0.37, **Table 3**). The VHI score of patients in the experimental group was significantly better than that in the control group (P < 0.05) at 24 h (2.89 ± 5.92 *vs*. 4.10 ± 6.31), 36 h (2.46 ± 5.06 *vs*. 3.43 ± 5.97), and 48 h (1.97 ± 2.74 *vs*. 2.74 ± 4.67), while the V-RQoL score was not significantly different between groups at any evaluation time point (P > 0.05, **Table 4**). There was no significant difference in swallowing function between the two groups of patients (P > 0.05, **Table 5**). No tetany of hands or feet was reported in either group and there was no statistically significant difference in the incidence of numbness in the hands, feet, and perioral area after surgery (P > 0.05, **Table 6**). There was no significant difference in the serum PTH and blood calcium after surgery (P > 0.05, **Table 7**).

**Table 2:** Comparison of pain scores between the two groups.

Variables	Evaluation time	Experimental group (n = 293)	Control group (n = 294)	Statistics	*P*
Incision pain	Time after arriving on the ward	1.26 ± 1.03	1.43 ± 1.17	*t* =1.854	0.064
	6 h	1.07 ± 0.80	1.23 ± 0.95	*t* =2.162	0.031**^※^**
	12 h	1.08 ± 0.64	1.20 ± 0.73	*t* =2.052	0.041**^※^**
	24 h	0.96 ± 1.01	1.03 ± 1.00	*t* =0.865	0.388
	36 h	0.84 ± 0.91	0.90 ± 0.89	*t* =0.831	0.406
	48 h	0.78 ± 0.84	0.84 ± 0.83	*t* =0.851	0.395
Sore throat	Time after arriving on the ward	1.52 ± 1.11	1.64 ± 1.14	*t* =1.303	0.193
	6 h	1.40 ± 0.85	1.59 ± 0.97	*t* =2.541	0.011**^※^**
	12 h	1.41 ± 0.67	1.55 ± 0.74	*t* =2.346	0.019**^※^**
	24 h	1.45 ± 0.98	1.50 ± 1.04	*t* =0.510	0.611
	36 h	1.27 ± 0.89	1.34 ± 0.99	*t* =0.910	0.363
	48 h	1.11 ± 0.78	1.22 ± 0.91	*t* =1.603	0.110

^※^indicates statistical significance; Pain score range: 0-10, with higher scores indicating more serious. The data are expressed as mean ± standard deviation.

**Table 3:** Comparison of PONV scores between the two groups.

Evaluation time	Experimental group (n = 293)	Control group (n = 294)	Statistics	*P*
Time after arriving on the ward	0.15 ± 0.55	0.17 ± 0.61	*t* =0.417	0.677
6 h	0.17 ± 0.49	0.17 ± 0.50	*t* =0.103	0.918
12 h	0.09 ± 0.19	0.14 ± 0.37	*t* =2.271	0.024**^※^**
24 h	0.08 ± 0.35	0.10 ± 0.43	*t* =0.864	0.388
36 h	0.02 ± 0.15	0.04 ± 0.22	*t* =1.292	0.197
48 h	0.02 ± 0.13	0.03 ± 0.16	*t* =0.834	0.404

^※^indicates statistical significance; PONV score range: 0-3, with higher scores indicating more serious. The data are expressed as mean ± standard deviation.

**Table 4:** Comparison of voice change scores between the two groups.

Variables	Evaluation time	Experimental group (n = 293)	Control group (n = 294)	Statistics	*P*
VHI score	Time after arriving the ward	5.78 ± 7.61	6.52 ± 8.09	*t* =1.135	0.257
	6 h	5.50 ± 5.82	6.18 ± 7.09	*t* =1.269	0.205
	12 h	5.52 ± 5.13	5.92 ± 5.06	*t* =0.947	0.344
	24 h	2.89 ± 5.92	4.10 ± 6.31	*t* =2.385	0.017**^※^**
	36 h	2.46 ± 5.06	3.43 ± 5.97	*t* =2.111	0.035**^※^**
	48 h	1.97 ± 2.74	2.74 ± 4.67	*t* =2.150	0.032**^※^**
V-RQoL score	Time after arriving on the ward	91.62 ± 13.45	90.13 ± 14.98	*t* =1.271	0.204
	6 h	92.24 ± 9.39	90.94 ± 12.46	*t* =1.429	0.153
	12 h	91.36 ± 11.09	91.42 ± 9.11	*t* =0.074	0.941
	24 h	95.62 ± 11.12	94.06 ± 12.06	*t* =1.628	0.104
	36 h	95.86 ± 10.87	94.34 ± 11.60	*t* =1.644	0.101
	48 h	96.22 ± 10.71	94.54 ± 11.47	*t* =1.834	0.067

^※^indicates statistical significance; VHI score range: 0-40, with higher scores indicating more serious; V-RQoL score range: 0-100, with lower scores indicating more serious. The data are expressed as mean ± standard deviation.

**Table 5:** Comparison of swallowing function between the two groups (n%).

Evaluation time	Stage of swallowing	Experimental group (n = 293)	Control group (n = 294)	*z*	*P*
6 h	I	276 (94.2%)	274 (93.2%)	0.475	0.635
	II	15 (5.1%)	20 (6.8%)		
	III	2 (0.7%)	0		
12 h	I	284 (96.9%)	283 (96.3%)	0.447	0.655
	II	9 (3.1%)	11 (3.7%)		
24 h	I	281 (95.9%)	277 (94.2%)	0.942	0.346
	II	12 (4.1%)	17 (5.8%)		
36 h	I	283 (96.6%)	282 (95.9%)	0.426	0.670
	II	10 (3.4%)	12 (4.1%)		
48 h	I	286 (97.6%)	285 (96.9%)	0.500	0.617
	II	7 (2.4%)	9 (3.1%)		

**Table 6:** Comparison of subjective symptom of numbness between the two groups (n%).

Evaluation time	Numbness	Experimental group (n = 293)	Control group (n = 294)	*χ^2^*	*P*
Time after arriving on the ward	Yes	219 (74.7%)	234 (79.6%)	1.958	0.170
	No	74 (25.3%)	60 (20.4%)		
6 h	Yes	240 (81.9%)	247 (84.0%)	0.459	0.512
	No	53 (18.1%)	47 (16.0%)		
12 h	Yes	260 (88.7%)	263 (89.5%)	0.078	0.793
	No	33 (11.3%)	31 (10.5%)		
24 h	Yes	238 (81.2%)	240 (81.6%)	0.016	0.916
	No	55 (18.8%)	54 (18.4%)		
36 h	Yes	235 (80.2%)	235 (79.9%)	0.007	1.000
	No	58 (19.8%)	59 (20.1%)		
48 h	Yes	233 (79.5%)	233 (79.3%)	0.007	1.000
	No	60 (20.5%)	61 (20.7%)		

**Table 7:** Comparison of changes in parathyroid function between the two groups.

Variables	Experimental group (n = 293)	Control group (n = 294)	Statistics	*P*
PTH(pmol/L)	2.44 ± 1.62	2.50 ± 1.73	*t* =0.362	0.718
Ca(mmol/L)	2.18 ± 0.16	2.19 ± 0.37	*t* =0.412	0.681

PTH:1.60-6.90 pmol/L; Ca:2.11-2.52 mmol/L.

## Discussion

In view of the low mortality and good tumor prognosis of thyroid cancer, the quality of life of patients after surgery is as important as disease control (23). Thus, how to reduce the incidence of postoperative complications while ensuring the thoroughness and safety of surgery and how to promote rapid rehabilitation of patients have become hot topics in the field of thyroid surgery (24, 25). The application of high-concentration oxygen in abdominal surgery, obstetric-gynecologic surgery, and tonsil surgery can significantly reduce the incidence of PONV, and some randomized controlled studies on the protective effect of sufficient high-concentration oxygen during the perioperative period show that inhalation of high-concentration oxygen can effectively reduce the incidence of incision infection after surgery (11–17). However, the specific mechanism remains unclear. Some researchers speculate that high-concentration oxygen can reduce the synthesis and release of inflammatory factors, which reduces the inflammatory response and immune response in the surgical area, thereby reducing edema at the surgical site and around the trachea, improving microcirculation, and improving nerve damage resistance (14, 15). Based on the above principles, it is conceivable that in thyroid surgery, high-concentration oxygen can reduce inflammatory and immune responses, reduce edema at the surgical site and around the trachea, improve the resistance of the recurrent laryngeal nerve and the superior laryngeal nerve to injury, and improve blood supply and oxygen supply to the parathyroid gland to thereby improve PONV, pain, voice change, and numbness of the hands, feet, and perioral area caused by impaired parathyroid function.

In this study, at 12 h after returning to the ward, the PONV score of the experimental group was better than that of the control group. Although the pain scores of the two groups were relatively low, we still observed that the postoperative pain degree of patients in the experimental group was lower than that in the control group at 6 and 12 h after returning to the ward. This indicates that high-concentration oxygen can improve the throat symptoms after thyroid surgery to a certain extent. Various throat symptoms after thyroid surgery are related to throat mucosal congestion and edema caused by intubation and extubation during general anesthesia, as well as aseptic inflammation and the immune response of soft tissue in the neck after surgery (4). High-concentration oxygen can effectively reduce edema caused by the inflammatory reaction at the surgical site of the neck (14, 15) to thereby improve the discomfort caused by the edema of the throat and alleviate the postoperative throat symptoms. The duration of oxygen use in this study was 6 h after returning to the ward, so the differences in the PONV score and pain score of the two groups of patients mainly appeared in the early stage after oxygen inhalation. The nonsignificant differences between groups at the later time points may be related to the fact that both the PONV score and the pain score were low. Another explanation may be that the inflammation and edema response of the throat spontaneously subsided over time. The gradual improvement of throat symptoms in both groups made it impossible to observe significant differences between the groups. However, Previous studies have shown supplemental oxygen does not reduce postoperative nausea and vomiting after thyroidectomy (18), but the study only included patients with benign thyroid lesions and their samples were small. Maybe that is the reason their research results are different from ours.

This study has shown that at 24 h, 36 h, and 48 h after returning to the ward, the VHI scores of patients in the experimental group were better than those in the control group, indicating that high-concentration oxygen can reduce the degree of voice change after thyroid surgery. Voice change or even loss of voice is one of the serious complications after thyroid cancer surgery. The traditional explanation is that laryngeal nerve function injury is the main cause of voice change (26). However, with the popularization of standardized surgical techniques and the application of intraoperative neuromonitoring in thyroid surgery, the probability of permanent injury to the recurrent laryngeal nerve has been greatly reduced (5). Voice changes may be related to a variety of causes, such as pain, vocal cord edema, decreased tension caused by anesthesia and intubation, traumatic vocal cord inflammation, laryngeal edema, and transient nerve damage caused by surgery (5, 27). With the increasingly frequent interpersonal communication, patients have a higher expectation for voice quality, which is related to the quality of life and work requirements of many patients, especially those who are engaged in language-related work. Moreover, some studies have shown that voice changes make patients more anxious than traditional complications do, such as hypocalcemia or neck scars (28). In terms of the prevention and treatment of recurrent laryngeal nerve injury in patients with thyroid cancer after surgery, the therapeutic use of glucocorticoids can promote the recovery of dysfunctional recurrent laryngeal nerves (29). The mechanism for this is that glucocorticoids can promote neurorecovery by eliminating neural edema. The reason why there was no difference in the V-RQoL score between the two groups of patients may be related to the fact that the voice changes during hospitalization did not have a significant impact on the patient’s voice-related quality of life.

In our study, the PONV, pain and sound scores of the two groups showed statistical differences in the early stage after initiation of high flow oxygen, but the statistically significant is low. It may be related to the generally low scores of these outcome indicators. As the sample size increases, this difference may become more pronounced. The experimental group has lower PONV, pain and sound scores upon arriving on the ward compared to the control group, and this trend is continued throughout the postop period. This result may be related to sampling error. The true clinical application of high-concentration oxygen is worthy of further study. In future studies, it is recommended that the trial be conducted in the hospital first, and then sampling in different regions and different levels of hospitals, so as to expand the sample size and increase the generalizability of the research results.

Patients with thyroid surgery will experience non-specific swallowing changes and discomfort after surgery (30), which may be caused by inflammation or edema resulting from repeated stimulation during surgery and postoperative cervical soft tissue damage (5), or by transient damage to the recurrent laryngeal nerve during surgery (6). Difficulty swallowing will affect patients’ early postoperative eating. In this study, the incidence of dysphagia in the two groups was low (less than 7%), and no significant difference between the groups was observed. Therefore, the protective effect of high-concentration oxygen on swallowing function after thyroid surgery needs to be further verified.

Decreased blood calcium caused by parathyroid function impairment is the most common complication after thyroid surgery. When blood calcium is reduced to a certain level, patients will develop numbness or convulsions of the hand, foot, and perioral area, and severe cases will involve systemic convulsions and even dyspnea. Traction and electrothermal injury during the surgery may cause tissue edema and vasospasms around the parathyroid glands, resulting in temporary hypoparathyroidism in patients (7). In this study, the two groups were similar in the incidences of numbness, serum PTH and serum calcium after surgery. These results may be related to the following aspects: First, the thyroid cancer patients in this study underwent routine lymph node dissection in the central compartment of the neck during surgery and concurrent autologous transplantation of the parathyroid glands, which may cause temporary functional impairment. High-concentration oxygen may not often reduce the occurrence of temporary hypoparathyroidism caused by the above surgical treatment. Second, in this study, physicians treated patients based on their subjective numbness and their postoperative serum PTH and blood calcium levels. Oral or intravenous calcium supplementation will not only affect the subjective numbness of patients but also affect serum PTH and blood calcium. Therefore, the protective effect of high-concentration oxygen on parathyroid function after thyroid surgery needs further verification.

This study had certain limitations. First, it was a single-center study with limited sample representativeness. In future studies, we recommended that samples be taken from hospitals in different regions and of different tiers to increase the generalizability of the results. Second, the degrees of PONV and pain are relatively mild in the thyroid cancer patients, and their subjective scores are generally low after surgery. Therefore, some negative results in this study may be related to sample size. Future studies with larger samples are needed. Third, this study did not evaluate the side effects of high-concentration oxygen application, which should be studied in the future.

## Conclusion

High-concentration oxygen can alleviate PONV and pain after thyroid surgery, with less severe voice changes potentially. However, its effects on swallowing function, and parathyroid function need to be further verified.

## Data Availabilty Statement

The original contributions presented in the study are included in the article/supplementary material, further inquiries can be directed to the corresponding author/s.

## Ethics Statement

This research protocol had been approved by the Biomedical Ethics Sub-Committee of West China Hospital, Sichuan University. The patients/participants provided their written informed consent to participate in this study. The studies involving human participants were reviewed and approved by This research protocol had been approved by the Biomedical Ethics Sub-Committee of West China Hospital, Sichuan University. The patients/participants provided their written informed consent to participate in this study.

## Author Contributions

The first authors of this manuscript are QZ and MC. All authors made substantial contributions to the conception and design, acquisition of data, or analysis and interpretation of data; took part in drafting the article or revising it critically for important intellectual content; gave final approval for the version for publication; and agree to be accountable for all aspects of the work. NN and JG are the guarantors and are directly responsible for the manuscript.

## Funding

This study was funding from research project of Sichuan Provincial Health Commission (project No. is 18PJ300).

## Conflict of Interest

The authors declare that the research was conducted in the absence of any commercial or financial relationships that could be construed as a potential conflict of interest.

## Abbreviations

PONV, postoperative nausea and vomiting; WHO, World Health Organization; VHI, Version Voice Handicap Index; V-RQoL, Voice-Related Quality of Life; PTH, Serum parathyroid hormone; LT, lobectomy of thyroid; TT, total thyroidectomy; UCND, unilateral central neck lymph node dissection; BCND, bilateral central neck lymph node dissection; T, tumor; N, node; M, metastasis.
